# Chronic Murine Typhoid Fever Is a Natural Model of Secondary Hemophagocytic Lymphohistiocytosis

**DOI:** 10.1371/journal.pone.0009441

**Published:** 2010-02-26

**Authors:** Diane E. Brown, Melissa W. McCoy, M. Carolina Pilonieta, Rebecca N. Nix, Corrella S. Detweiler

**Affiliations:** 1 Department of Molecular, Cellular and Developmental Biology, University of Colorado, Boulder, Colorado, United States of America; 2 Paleontology Section, Museum of Natural History, University of Colorado, Boulder, Colorado, United States of America; Charité-Universitätsmedizin Berlin, Germany

## Abstract

Hemophagocytic lymphohistiocytosis (HLH) is a hyper-inflammatory clinical syndrome associated with neoplastic disorders especially lymphoma, autoimmune conditions, and infectious agents including bacteria, viruses, protozoa and fungi. In both human and veterinary medicine, hemophagocytic histiocytic disorders are clinically important and frequently fatal. HLH in humans can be a primary (familial, autosomal recessive) or secondary (acquired) condition, with both types generally precipitated by an infectious agent. Previously, no mouse model for secondary HLH has been reported. Using *Salmonella enterica* serotype Typhimurium by oral gavage to mimic naturally-occurring infection in Sv129S6 mice, we characterized the clinical, hematologic and morphologic host responses to disease thereby describing an animal model with the clinico-pathologic features of secondary HLH as set forth by the Histiocyte Society: fever, splenomegaly, cytopenias (anemia, thrombocytopenia), hemophagocytosis in bone marrow and spleen, hyperferritinemia, and hypofibrinogenemia. Disease severity correlates with high splenic and hepatic bacterial load, and we show disease course can be monitored and tracked in live animals. Whereby secondary HLH is known to occur in human patients with typhoid fever and other infectious diseases, our characterization of a viable natural disease model of secondary HLH offers an important means to elucidate pathogenesis of poorly understood mechanisms of secondary HLH and investigation of novel therapies. We characterize previously unreported secondary HLH in a chronic mouse model of typhoid fever, and novel changes in hematology including decreased tissue ferric iron storage that differs from classically described anemia of chronic disease. Our studies demonstrate *S*. Typhimurium infection of mice is a natural infectious disease model of secondary HLH that may have utility for elucidating disease pathogenesis and developing novel therapies.

## Introduction

Hemophagocytic lymphohistiocytosis (HLH), an inflammatory syndrome characterized by over-activation of macrophages and T lymphocytes, can be triggered by diverse eukaryotic, bacterial (especially intracellular), and viral pathogens [1]–[5]. HLH mortality rates can reach 50–90% in part due to late recognition and delayed onset of treatment [1], [4]. Patients with clinico-pathological characteristics of HLH are given differential diagnoses that include Macrophage Activation Syndrome, Hemophagocytic Histiocytosis, and Hemophagocytic Syndrome. The Histiocyte Society has endeavored to improve clinical recognition and resolve nomenclature issues by establishing a standard for HLH diagnostic criteria [5]. Veterinary literature has reflected similarly variable nomenclature for animals with hemophagocytic histiocytic disorders [6]–[10]. Regardless of primary diagnosis, the 2004 HLH diagnostic criteria require a patient have five of eight characteristics: hemophagocytic macrophages in bone marrow, spleen, and/or lymph nodes; two of three cytopenias (anemia, neutropenia, and/or thrombocytopenia); splenomegaly; hyperferritinemia; hypertriglyceridemia and/or hypofibrinogenemia; fever; high soluble CD25; and low natural killer (NK) cell activity [5].

HLH can be primary (familial, fHLH) or secondary (sHLH). Familial HLH is autosomal recessive, typically diagnosed in infants or children, fatal if untreated [2], [4], and generally precipitated by infectious disease [3], [5], [11]. Thirty to seventy percent of fHLH cases are associated with genetic mutations that cause NK and/or CD8^+^ T cell defects [5]. Four fHLH mouse models have been described [12]–[15]; three have spontaneous or genetically engineered mutations in *Prf1*, *Unc13d* or *Rab27a*, and fHLH is triggered by viral infection. A fourth model is in mice deficient for asparaginyl endopeptidase (AEP, legumain) [15]. In summary, current mouse models of HLH involve genetic lesions and are models of fHLH.

Secondary HLH occurs in all age groups and is associated with infections across classes, malignancies especially lymphoma [1], [3], and autoimmune disorders, absent any known underlying genetic defect [2]. HLH often has a nonspecific clinical presentation, and although pathophysiologically distinct, is difficult to clinically differentiate from sepsis [1], [2], [16], underlining the clinical importance of the Histiocyte Society's diagnostic criteria [5]. Difficulty in quantifying prevalence of sHLH is partly due to the diversity of underlying primary diseases, and evidence suggests it is likely under-diagnosed [2], [4], [17]. A fatality rate of 40% is reported for sHLH cases without appropriate immuno-modulatory therapy [4]. Standardized diagnostic criteria should continue to improve recognition and yield more accurate prevalence statistics [5]. There are no reported mouse models of sHLH.

Data herein demonstrate that *Salmonella enterica* serotype Typhimurium (*S*. Typhimurium)-infected mice have clinico-pathological features of sHLH. Laboratory mice infected with *S*. Typhimurium model human typhoid fever which is caused by *Salmonella enterica* serovars Typhi and Paratyphi A, B and C [18]. Bacteria establish a chronic systemic infection in macrophages of Peyer's patches, mesenteric lymph nodes (MLN), spleen, liver and gall bladder [19]–[21]. Much of *S*. Typhimurium research has focused on fatal acute infections in mice compromised for innate immunity, generally Balb/c, C57BL/6, or DBA/1 strains, which are Slc11a1/Nramp1^−/−^ [22], [23]. However, in immunocompetent mice (Sv129S6, Slc11a1/Nramp1^+/+^) *S*. Typhimurium causes persistent systemic infection that most mice survive [19]. Both murine and human typhoid fever can result in a subclinical chronic carrier state [18], [21]. Bacteria are found within macrophages during both acute and chronic infection [19], [24], [25], and cytokines including IL-1β and IL-18 are produced during pro-inflammatory caspase-1 dependent programmed cell death (pyroptosis) [26]. Hemophagocytic macrophages are a feature of both human typhoid fever [27]–[30] and HLH [5]. Importantly, *S*. Typhimurium replicates preferentially within cultured hemophagocytic macrophages in the Sv129S6 mouse model [24]. Here we demonstrate that Sv129S6 mice infected with *S*. Typhimurium acquire the clinico-pathological characteristics of HLH (Table 1), thus establishing an animal model for sHLH.

**Table 1:** Clinico-pathologic features of HLH in *S.* Typhimurium-infected mice.

	One-week PI	Three-weeks PI	Six-weeks PI	Ten-weeks PI
‡Increased hemophagocytic macrophages (bone marrow and/or spleen)	5/6 mice	4/6 mice	6/7 mice	2/2 mice
‡Fever		Days 17–23*		
‡Splenomegaly, % of body weight: median (range),fold difference from C	2X* (2-3X)6/6 mice	3X (1-14X)5/6 mice; 10/10 mice†	3X* (1-5X)6/7 mice	3X (1-3X)1/2 mice
‡Cytopenias: thrombocytopeniaC range = 9.2–10.5×10^5^/µL anemiaC range = 53–55 (HCT%)	2/6 mice(7.7–11.1)1/6 mice*(48–53)	5/6 mice*(0.9–9.8)2/6 mice(29–53)	3/6 mice*(5.0–9.3)0/6 mice(51–56)	0/2 mice(10.6–10.7)0/2 mice(51–55)
‡HypofibrinogenemiaC range (200–400 mg/dl)	0/6 mice	2/6 mice(100)	2/6 mice(100)	0/6 mice
‡HyperferritinemiaC range (699–1260 µg/L)	No	2/6 mice;3/10 mice†(1425–6140)	No	No
Range CFU/g: spleen	10^2^–10^4^	10^4^–10^5^	10^3^–10^4^	10^1^–10^2^
liver	10^2^–10^4^	10^3^–10^4^	10^2^–10^3^	None detected
§Clinical neurological signs	7/21 mice- onset Day 5–12	6/21 mice – signs gone by Day 18	1/21 mice- early death Day 37	None
^Π^Chronic active hepatitis	Acute hepatitis 5/6 mice	Chronic active hepatitis 5/6 mice	Chronic active hepatitis 7/7 mice	Chronic active hepatitis 1/2 mice

PI indicates post-oral infection with 2.0×10^9^ CFU *Salmonella enterica* serotype Typhimurium; C indicates mock-infected control mice.

* *P*<0.05, Student's *t*-Test.

† Independent experiment, same bacterial dosing and range for splenic bacterial CFU results.

‡ Formal diagnostic criteria for HLH per the Histiocyte Society guidelines [5].

§ Consistent with a diagnosis of HLH, and ^Π^ strong supportive evidence for HLH [5].

## Results

**Figure 1:**
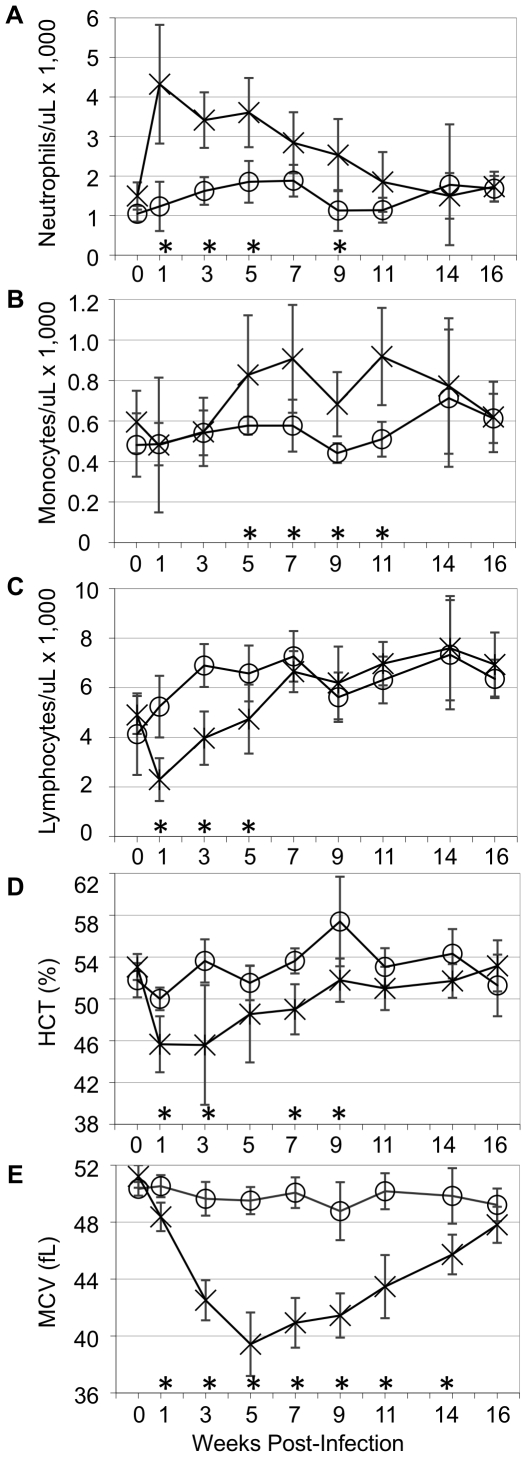
Hematology of *S.* Typhimurium-infected mice: acute, then chronic active inflammatory response; microcytic anemia, persistent microcytosis. Mice were orally gavaged with 9.1×10^8^ CFU of *S*. Typhimurium (n = 8) or sterile PBS (n = 7). Complete blood counts were monitored over 16 weeks. X  =  *S*. Typhimurium-infected mice; circle  =  mock-infected control mice. Mean and standard deviation are shown. (A) neutrophils, (B) monocytes, (C) lymphocytes, (D) hematocrit (HCT), (E) mean cell volume (MCV). *P<0.05 (Student's *t*-test).

Our findings of neurological disease, splenomegaly and inflammatory lesions are consistent with prior descriptions of murine *S*. Typhimurium infection [18], [19], [24], [25], [31]–[33], [21]. Additionally, we describe detailed hematopoietic responses over the course of infection that characterize a) a non-lethal method to monitor *S*. Typhimurium infection via complete blood count (CBC), b) features of a secondary HLH syndrome, and c) responsive hematopoiesis with alterations in body iron that differ from anemia of inflammatory (or chronic) disease (ACD). Hematology and clinico-pathologic findings in *S*. Typhimurium-infected Sv129S6 mice are summarized in Figure 1 (16-week study), and Tables 1 and 2 (10-week study).

**Table 2:** Hematologic parameters of *S.* Typhimurium-infected mice.

Parameter	Week 1		Week 3		Week 6		Week 10	
	C, n = 4	I, n = 6	C, n = 4	I, n = 6	C, n = 3	I, n = 6	C, n = 1	I, n = 2
WBC×10^3^/µl	5.73 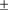 1.61	5.32 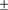 1.82	7.23 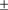 1.93	5.32 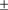 2.01	7.03 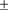 1.62	8.13 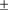 1.99	6.8	14.8, 6.7
RBC×10^6^/µl	9.79 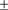 0.37	9.70 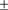 0.25	9.79 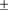 0.31	9.20 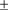 1.83	9.64 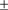 0.32	11.42* 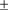 0.25	8.86	11.43, 9.42
Neutrophils x10^3^/µl	0.50 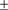 0.20	1.35* 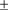 0.61	0.75 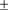 0.21	1.25* 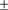 0.43	0.63 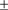 0.23	1.82* 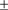 0.63	0.40	4.00, 0.60
Monocytes x10^3^/µl	0.07 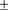 0.06	0.17 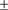 0.10	0.10 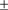 0	0.23* 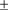 0.05	0.10 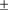 0	0.43* 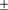 0.10	0.10	0.50, 0.10
Lymphocytes x10^3^/µl	4.73 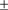 1.34	3.57 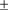 1.84	6.28 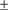 1.37	3.72 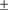 2.24	6.00 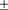 1.68	5.70 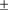 2.02	6.10	10.10, 5.80
HCT, %	53 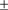 0.6	51* 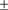 1.6	53 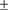 1.7	46 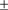 11.3	53 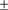 1.2	54 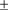 1.8	51	55, 51
MCV, fl	54.0 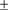 2.0	52.0 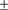 0.9	54.3 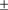 1.3	49.7 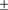 4.8	55.7 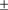 1.5	47.3* 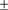 1.0	58.0	48.0, 54.0
MCVr, fl	59.3 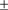 1.5	56.3 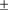 3.1	59.3 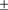 0.5	52.8* 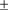 5.4	62.0 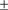 1.0	55.8* 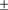 1.7	62.0	59.0, 63.0
MCHC, g/dl	31.0 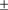 0	31.5 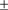 0.5	31.3 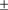 0.5	30.0 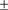 1.8	30.0 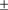 1.0	30.0 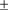 0.6	30.0	30.0, 31.0
RDW	12.4 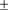 0.4	12.7 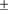 0.2	12.5 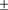 0.2	16.7 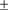 4.9	12.7 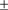 0.1	14.6* 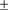 1.5	12.6	14.3, 13.0
Platelets x10^6^/µl	1.02 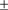 0.03	1.00 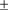 0.14	0.92 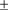 0.06	0.54* 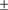 0.33	1.05 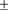 0.06	0.79* 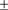 0.16	0.87	1.06, 1.07
MPV, fl	7.3 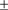 0.3	7.1 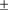 0.2	7.3 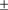 0.3	7.2 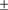 1.0	7.7 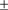 0.2	8.2 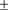 0.6	6.7	7.1, 6.7
Reticulocytes x10^6^/µl	0.48 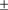 0.07	0.41 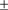 0.18	0.40 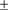 0.07	0.71 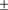 0.40	0.44 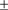 0.03	0.41 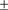 0.06	0.57	0.54, 0.64
CHr, pg	17.9 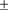 0.4	16.7* 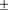 1.0	17.7 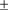 0.1	15.0* 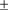 2.3	17.4 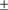 0.2	15.5* 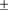 0.4	17.4	15.817.8
Serum Iron µg/dl	262 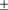 10	297 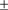 124	267 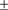 26	219 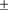 89	269 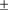 30	266 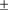 40	222	202, 280

Twenty-one mice were orally gavaged with 2.0×10^9^ CFU of *S*. Typhimurium (I) and 13 mice with sterile PBS as mock-infected controls (C). No hematological data were obtained from one early death mouse. Complete blood counts (CBC) and serum iron were measured at weeks 1, 3, 6, and 10 post-infection. Data presented as mean 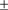
1 standard deviation, except 10 weeks where data from all mice shown.

WBC indicates white blood cells; RBC, red blood cells; HCT, hematocrit; MCV, mean cell volume; MCVr, mean cell volume reticulocytes; MCHC, mean cell hemoglobin concentration; RDW, red cell distribution width; MPV, mean platelet volume; and CHr, mean cell hemoglobin reticulocytes.

* *P*<0.05, Student's *t*-Test.

### Clinical and Hematological Findings

#### Fever and neuropathy

Infected mice developed slight fevers and clinical neurological signs including ataxia, head tilt, uni-directional circling when ambulating, and uni-directional rotatory spinning when lifted by the tail in seven of twenty-one mice (Table 1), and two of eight mice (16-week study). Mice with the most severe neurological signs failed to gain weight and accounted for the single early death in each study. While only fever is a formalized diagnostic criterion for HLH, both fever and neurological disease are common to HLH [1], [3], [2], [34].

#### Inflammatory leukogram

Hematological findings included acute and ongoing response to infection characterized by immediate onset of neutrophilia (Figure 1A; Table 2) and later onset (3–5 weeks post-infection) of monocytosis (Figure 1B; Table 2). Rare band neutrophils were also seen. Lymphopenia (Figure 1C; Table 2) was consistent with a stress response from inflammatory disease and/or tissue infiltration by lymphocytes [1], [35]. Up to 30% of lymphocytes had vacuolated cytoplasm. Thus, an acute inflammatory response transitioning to chronic active inflammation was demonstrated.

#### Responsive microcytic anemia

**Figure 2:**
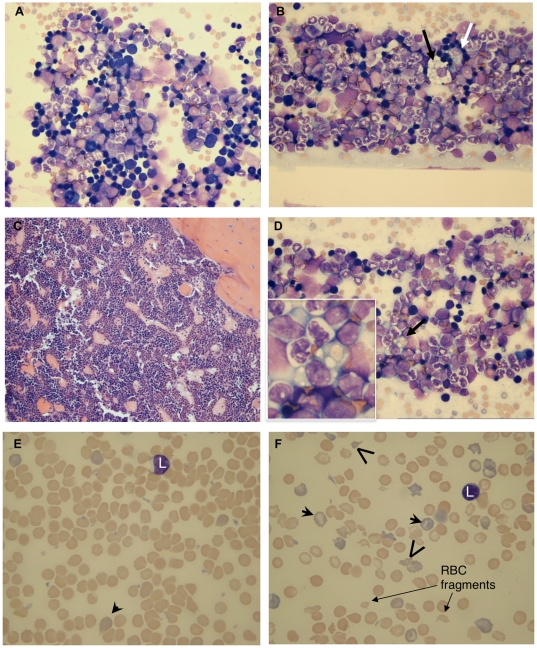
*S*. Typhimurium-infected mice have increased hemophagocytic macrophages, myeloid hyperplasia, regenerative microcytic anemia and erythrocyte fragmentation. (A) Control bone marrow cytology, 3 weeks post mock-infection. (B) Infected mouse bone marrow cytology, 3 weeks post-infection; myeloid hyperplasia with increased blasts and monocytes, hemophagocytic macrophage (white arrow), and foamy macrophage (black arrow). (C) Bone marrow histology, 6 weeks post-infection; hypercellularity with myeloid hyperplasia. (D) Mouse bone marrow cytology, 3 weeks post-infection; erythrocyte (arrow and inset) within hemophagocytic macrophage. (E) Control mouse blood film, 3 weeks post mock-infection. (F) Blood film from highly infected mouse, 3 weeks post-infection; marked erythrocyte anisocytosis, increased polychromasia and markedly fragmented erythrocytes (mature and polychromatophilic). Polychromatophilic erythrocytes (arrow-heads), fragmented polychromatophilic erythrocytes (carats), fragmented erythrocytes (arrows), L = small lymphocyte. Wright stain (A, B, D, E, F). H & E stain (C). Original magnifications 500× (A–B, D), 200× (C), or 1000× (E–F).

Infected mice had early onset slight to moderate microcytic anemia which was most severe at three weeks post-infection, correlating with highest bacterial tissue loads and most severe splenomegaly (Figure 1D–E; Table 2). Even after hematocrits (HCT) had recovered, microcytosis of erythrocytes (Figures 1D–E; Table 2) and reticulocytes (Table 2) persisted (demonstrated as decreased mean cell volume, MCV and MCVR, respectively). A marked regenerative erythroid response, measured by increased reticulocyte counts, polychromasia (Tables 1–2; Figure 2F) and red cell distribution widths (RDW) (Table 2), was present in highly infected mice. Reticulocytes were hypochromic (decreased CHr) at all time points (Table 2). These changes occurred together with hypoferremia in all but one infected mouse at three weeks post-infection; this mouse had an appropriate macrocytic regenerative response consistent with its normal serum iron concentration (Table 2). Erythrocytosis, which can occur with microcytic anemia [36], [37], was present from weeks 6–10 (Table 2, 10-week study), and 5 – 14 (*P*<0.05; 16-week study). Increased RDW at week six (Table 2) and weeks 3–14 (*P*<0.05; 16-week study) demonstrated erythrocyte anisocytosis. Blood film review confirmed erythrocyte anisocytosis, microcytosis and polychromasia (Figure 2E–F). Marked fragmentation (schistocytes) of mature and polychromatophilic erythrocytes (10–12% of cells affected) occurred at three weeks in highly infected mice (Figure 2F). Schistocytes were rare (∼1%) at six weeks, and absent at 10 weeks post-infection. These data represent regenerative microcytic anemia and altered body iron status in highly infected mice.

#### Thrombocytopenia

Thrombocytopenia present from weeks 1–10 (Tables 1, 2) was most severe in highly infected mice at three weeks post-infection. Increased mean platelet volume (MPV) (Table 2) indicated increased thrombopoiesis in response to thrombocytopenia [35], and circulating macro-platelets were confirmed by blood film review; increased MPV was also present at weeks 1–11 (P<0.05) in the 16-week study (data not shown). Thus, *S*. Typhimurium infection in live animals can be monitored by CBC, and bi-cytopenia (anemia and thrombocytopenia), characteristic for HLH [5], was demonstrated in highly infected mice.

### Bacterial Tissue Loads and Splenomegaly

Serum IgG titers to *Salmonella* O-Antigen, measured in the 16-week study, were 100–1,000 fold higher than mock-infected control from weeks 5–16 post-infection (data not shown). At 16-weeks post-infection, there was no recoverable *S*. Typhimurium in cecum, MLN, liver or spleen in five of eight mice, including two of five mice with high serum IgG. In the 10-week experiment (Table 1), bacterial colony forming units per gram (CFU/g) of liver and spleen was greatest at three weeks post-infection, consistent with the time-point of most severe HLH pathology (Tables 1–2), and decreased by six and 10 weeks post-infection.

Splenomegaly was present in six of seven infected mice at the conclusion of the 16-week study (*P*<0.05). Hepatosplenomegaly occurred at all time points in *S*. Typhimurium-infected mice in the 10-week study (Table 1). By comparison, livers of infected mice were 1.2 to 1.6 fold larger (median weight compared to body weight) than mock-infected controls overall (*P*<0.05 at one and three weeks post-infection). Mice with the highest splenic bacterial loads (10^5^ CFU/g at three weeks post-infection) had the largest spleens, and also the highest liver tissue loads (>5×10^4^ CFU/g) and largest livers. An inverse correlation occurred between spleen weight, and HCT and platelet counts; infected mice with the greatest degree of splenomegaly had the lowest HCT and platelet counts. Importantly, splenomegaly persisted beyond the presence of measurable CFU of *S*. Typhimurium, and splenic bacterial load and splenomegaly correlated with the most severe cytopenias.

### Bone Marrow Cytopathology, Tissue Histopathology and Iron Studies (10-Week Experiment)

#### Bone marrow myeloid hyperplasia and splenic extramedullary hematopoiesis (EMH)

**Figure 3:**
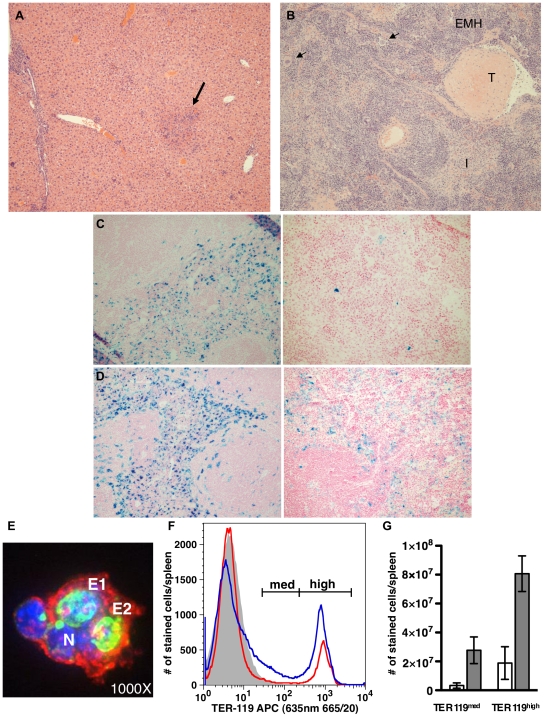
*S.* Typhimurium-infected mice have tissue inflammation and thrombosis, increased hematopoiesis, and decreased splenic iron. (A) Mouse liver, 6 weeks post-infection; inflammation and necrosis (arrow). (B) Mouse spleen, 3 weeks post-infection; extramedullary hematopoiesis (EMH; arrow, megakaryocytes), histiocytic infiltration (I) throughout the red pulp, and thrombus (T). H&E stain (A, B). (C) Spleen, mock-infected (left) and infected mouse (right), 3 weeks post-infection; markedly decreased ferric iron staining in red pulp. (D) Spleen, mock-infected (left) and infected mouse (right), 6 weeks post-infection; markedly decreased splenic ferric iron in red pulp. Perl's Prussian Blue stain (C, D). (E) Hemophagocytic macrophage in mouse spleen 3 weeks post-infection that had 10-fold more macrophages and 43-fold more 6N+ macrophages than control mouse spleen. CD11b (red), DAPI (blue), TER119 (green). N  =  endogenous macrophage nucleus, E1  =  nucleated erythrocyte, E2  =  non-nucleated erythrocyte. Confocal fluorescent micrograph. (F) Representative histogram overlay of TER119 expression on DAPI+ splenocytes from a mock-infected (red) and infected mouse (blue) 3 weeks post-infection. Filled gray histogram corresponds to the isotype control. The infected mouse had 11.5-fold more TER119^med^ pro-erythroblasts and 5.5-fold more TER119^high^ erythroblasts than the mock-infected mouse. (G) Mean numbers of TER119^med^ and TER119^high^ splenocytes from three mock-infected (white bars) and four infected (gray bars) mice. Mean number of TER119^med^ pro-erythroblasts per spleen increased 6.8-fold in infected mice, while the mean number of TER119^high^ cells, corresponding to all nucleated erythroblasts subsequent to the pro-erythroblast stage [40], increased 3.6-fold. (*P*<0.05) Error bars = SD. Original magnifications 100× (A–B), 200× (C–D), and 1000× (E).

Bone marrows were hypercellular (Figure 2C), and increased splenic erythroid, myeloid and megakaryocytic EMH (Figure 3B) was observed at all time points post-infection, consistent with inflammation and murine responsive hematopoiesis [35], [38]. While erythroid elements were present, bone marrow hypercellularity was primarily due to increased granulopoiesis (myeloid hyperplasia) with increased numbers of blasts (myeloblasts and monoblasts), monocytes and morphologically benign histiocytes (Figure 2A–B). Increased numbers of blasts in the bone marrow at three weeks post-infection (maturation left shift) were characteristic for accelerated production of granulocytic and/or monocytic precursors in response to ongoing infection (Figure 2B). While differentiating myeloblasts from monoblasts by morphology alone on review of bone marrow cytology is difficult [39], a corresponding increase in blood monocyte counts (Table 2) indicated that a proportion of the blasts were of monocytic origin. Increased granulocytic and monocytic ring forms in bone marrow and blood films (not shown) were consistent with accelerated hematopoiesis in mice [35], [39]. These findings correlate with the CBC results of neutrophilia and monocytosis, with ongoing inflammatory disease and neutrophil recruitment to tissues (Table 2; Figure 3A–B), and increased splenic EMH (Figure 3B).

To further evaluate erythropoiesis, enlarged spleens from Salmonella-infected mice were analyzed by flow cytometry three weeks after infection. Results from three replicate experiments showed increased numbers of erythroblasts compared to controls as measured by TER119 staining [40] (Figure 3F–G). Thus, increased splenic erythropoiesis was substantiated and quantified by flow cytometric analysis. Bone marrow and splenic pathology in *S*. Typhimurium*-*infected mice indicated accelerated hematopoiesis in response to active and ongoing inflammatory disease, anemia and thrombocytopenia.

#### Bone marrow hemophagocytic macrophages

Increased numbers of hemophagocytic macrophages in the bone marrow or spleen are consistent with HLH [5] (Table 1). Review of bone marrow films demonstrated increased hemophagocytic macrophages in mice at all time points post-infection (Table 1; Figure 2B,D), from occasional (1–3/slide) to numerous (1/1–2 high power field) compared to mock-infected control mice (0–1/slide). Finding two hemophagocytic macrophages per slide is considered significant when reviewing bone marrow cytology for human HLH [28]. Increased hemophagocytosis, present in spleens of infected mice examined by histopathology, was also demonstrated by confocal microscopy via TER119 staining of nucleated and non-nucleated erythrocytes within the cytoplasm of CD11b^high^, GR1^low^ macrophages (Figure 3E). Consistent with sHLH and typhoid fever [28], [29], *S*. Typhimurium-infected mice have increased numbers of hemophagocytic macrophages in bone marrow and spleen.

#### Liver and spleen histopathology, thrombosis and hypofibrinogenemia

Tissue inflammation and thrombosis occurred in *S*. Typhimurium-infected mice. Acute multifocal to coalescing neutrophilic hepatitis with necrosis was present at one week post-infection. At subsequent time-points, chronic active inflammation was characterized by multifocal to diffuse lympho-histiocytic infiltration with a neutrophilic component, and multifocal areas of hepatocellular necrosis (Figure 3A). Continuous recruitment of neutrophils was evident in all infected mice. Chronic active hepatitis, while not a formalized diagnostic criterion for HLH, is consistent with the described liver lesion in human HLH [5]. Splenic lesions included multifocal histiocytic infiltration and hemophagocytosis, with marked expansion of the red pulp by EMH and morphologically benign histiocytes at all timepoints (Figure 3B). Lymphoid follicular disruption with depletion of the white pulp was also present (Figure 3B), particularly at six and 10 weeks post-infection, consistent with a previously described change in mice with marked EMH in response to anemia [36], and splenic pathology in people with HLH [28]. Hepatic microthrombi were present in two of six mice at one and six weeks post-infection, while marked thrombosis was present in the spleen (Figure 3B) and liver (not shown) in two of six mice at three weeks post-infection. These findings correlated with plasma hypofibrinogenemia in the highly infected mice at three and six weeks post-infection (Table 1). Marked splenic and hepatic thrombosis also occurred in the early death mouse at six weeks post-infection. Masson's staining was negative for fibrosis in liver and spleen sections at all time points (not shown). Thus, persistent hepatosplenomegaly as a result of ongoing inflammatory lesions and EMH in the spleen was present in *S*. Typhimurium-infected mice, and tissue thrombosis correlated with hypofibrinogenemia, an HLH diagnostic criterion [5].

#### Hyperferritinemia, hypoferremia, decreased tissue iron staining

Serum iron was decreased at one (two of six mice) and three (four of six mice) weeks (Table 2) post-infection compared to control mice. Hyperferritinemia occurred in 2/6 and 3/10 mice in separate experiments at three weeks post-infection (Table 1). Mice with splenic bacterial loads >10^5^ CFU/g and the largest degree of splenomegaly had the most marked hyperferritinemia (>4,300 µg/L) (Table 1). By six weeks post-infection serum ferritin and iron concentrations were similar to controls although splenomegaly and erythrocyte microcytosis persisted (Tables 1–2). Spleen was the predominant iron storage organ in control mice at all time points where staining intensity for ferric iron in the splenic red pulp (3C–D) was two to four-fold greater than control livers, and four to eight-fold greater than control bone marrows. This is consistent with spleen being the major site for iron storage and EMH [35], [33] compared to liver in wild type, healthy mice. Overall, decreased tissue staining for hemosiderin (ferric iron) occurred at all time points post-*S*. Typhimurium-infection in mouse spleen (Figure 3C–D), liver and bone marrow (not shown) compared to mock-infected control mice. In addition, another feature of HLH, hyperferritinemia, was demonstrated in mice with high tissue bacterial loads, and significant changes in body iron status occurred post-infection with *S*. Typhimurium.

To summarize results, evaluations of blood, bone marrow, liver and spleen of *S*. Typhimurium-infected mice demonstrated active and ongoing hematopoiesis, inflammatory disease, and six of eight characteristics of sHLH in highly infected mice.

## Discussion

Infection of Sv129S6 wild-type mice with *S*. Typhimurium via the natural oral route precipitates, as expected [18], [19], an acute inflammatory response followed by subacute to chronic inflammatory disease. While the inflammatory response is consistent with prior descriptions of murine typhoid fever [18], [19], [23], [31], new to this infectious disease model are clinico-pathologic features of HLH (Table 1) demonstrated by increased hemophagocytic macrophages in bone marrow and spleen, fever, hepatosplenomegaly, bi-cytopenia (anemia, thrombocytopenia), hypofibrinogenemia, and hyperferritinemia (>1400 µg/L). At three weeks post-infection, the most severely infected mice with the highest bacterial CFU/g of spleen meet six of eight of the HLH diagnostic criteria, five of which are needed to diagnose HLH in humans [5]. These data substantiate a novel clinical mouse model for sHLH.

While a diagnosis of HLH cannot be solely based on finding hemophagocytic macrophages, an increased number especially in bone marrow cytology (Figures 2B,D) and spleen (Figure 3E) is a classic characteristic of HLH [5], [28], and also occurs with human typhoid fever [3], [28], [29]. In a prior study demonstrating hemophagocytic macrophages in livers of infected mice, *S*. Typhimurium was shown to survive and replicate in tissue culture hemophagocytic macrophages [24]. Erythrophagocytic macrophages have been shown to express heme-oxygenase 1 (HO-1) in human sepsis cases [41], and inhibit pro-inflammatory cytokine production in cultured mouse bone marrow cells (Slc11a1/Nramp1^+/+^) [42], thereby suggesting an anti-inflammatory role. Increased serum HO-1 also occurs in cases of sHLH in correlation with hyperferritinemia [43]. Whether HO-1 facilitates *S*. Typhimurium survival in hemophagocytic macrophages is unknown, however, a plausible hypothesis is that intracellular bacterial access to iron released during heme breakdown following erythrophagocytosis provides *S*. Typhimurium with an essential survival nutrient. Thus, hemophagocytic macrophages may provide a survival niche for *S*. Typhimurium contributing to the subclinical chronic carrier state important in people and mice with typhoid fever, as well as the pathology of cytopenias, including anemia and thrombocytopenia, in *Salmonella*-infected mice. Hemophagocytic macrophages may have an anti-inflammatory effect, provide *S*. Typhimurium a survival niche, and contribute to development of cytopenias, all of which could help elucidate mechanisms of sHLH disease pathogenesis in this natural infectious disease model.

Activated macrophages are integral to the development of HLH [1], [2]. Secretion of plasminogen activator by activated macrophages contributes to the coagulopathies that occur in HLH [2], [28]. Indeed, disseminated intravascular coagulation (DIC) has been reported in humans with HLH [1], [3], [44] or typhoid fever [45], and highly infected mice in our study had characteristics of DIC including thrombocytopenia, (Figure 1; Tables 1–2) hypofibrinogenemia, tissue thrombosis (Figure 3B), and schistocytes in the blood (Figure 2F). In humans, severe hyperferritinemia in HLH correlates with death [1] and is useful as a diagnostic marker for HLH [4], [5], [43], [44]. Serum ferritin levels are highest (>12,000 µg/L) in human histiocytic disorders in which the patients also have DIC [44]. Approximately 30% of *S*. Typhimurium-infected mice in our studies had hyperferritinemia that was greatest (>4,300 µg/L) in mice with most severe splenomegaly and splenic bacterial CFU (Table 1). Hyperferritinemia has also been reported in canine and feline histiocytic disorders [6], [9], and in acute *S*. Typhimurium infection in mice [23], and should alert both human and veterinary clinicians to the possibility of hemophagocytic histiocytic disorders. These observations collectively indicate activated macrophages play a key role in the pathophysiology of *S*. Typhimurium-induced sHLH.

The pathogenesis of bi-cytopenia in this mouse model is likely multifactorial. Adequate to increased megakaryocytes in bone marrow (Figure 2C) and spleen (Figure 3B) make lack of platelet production an unlikely cause for thrombocytopenia. Platelet sequestration within enlarged spleens may have contributed to thrombocytopenia. Additionally, increased platelet destruction by hemophagocytosis or platelet consumption from DIC [35] may have occurred in highly infected mice (Table 1). Also, regardless of bacterial tissue load, *S*. Typhimurium-infected mice developed erythrocyte microcytosis, and highly infected mice developed a regenerative microcytic anemia that differed from ACD by a demonstrated lack of tissue iron sequestration [35], [46] (Figure 3C–D) even in mice with hyperferritinemia. Our findings of increased erythropoiesis and hemophagocytosis differ from the non-regenerative anemia previously described in acute, fatal *S*. Typhimurium infection in genetically susceptible mouse strains [23]. The pathogenesis of microcytosis in *S*. Typhimurium-infected mice in our studies may involve several mechanisms including iron deficiency [37], [47] or fragmentation of erythrocytes [48] known to occur with both iron deficiency [37] and DIC. Erythrocyte fragmentation was severe enough to decrease MCV only in highly infected mice that also had evidence of DIC and low serum iron concentration. Persistent microcytosis following recovery of anemia and reduced tissue iron staining indicate iron stores were mobilized to support erythropoiesis and/or sequestered in ferritin as a result of increased heme breakdown by macrophages. As iron stores were depleted, ongoing accelerated erythropoiesis could have resulted in a progressive decrease in MCV as in iron deficiency. Another hypothesis for persistent microcytosis is altered iron metabolism. Changes in iron metabolism have been demonstrated in response to acute *S*. Typhimurium infections in susceptible strains of mice [23] and in tissue culture macrophages [49]. Functional iron deficiency may be a mechanism for controlling infection by limiting microbial access to iron. Decreased intracellular iron content of *S*. Typhimurium-infected macrophages [49] is consistent with reduced tissue iron storage in macrophages (Figure 3C–D) and differs from ACD. These findings suggest iron was mobilized from tissues to support stimulated erythropoiesis in the face of cytopenias, because of increased ferritin synthesis, and alternatively, or in addition, there may have been efflux of iron from macrophages presumably via the iron exporter ferroportin to reduce intracellular *S*. Typhimurium access to iron, a mechanism previously described [49]. Thus, the hematologic effects and depleted tissue iron storage in chronic *S*. Typhimurium-infected mice differentiate it from ACD and from acute fatal murine salmonellosis, and this model provides an opportunity to study mechanisms for anemia in HLH and altered iron trafficking in response to infection with *S*. Typhimurium.

The mouse strain chosen for this study was important given the effect of murine genetic variability on iron homeostasis [50], hematology [23], [35], [38] and *S*. Typhimurium resistance [19], [22]. Sv129S6 mice, intact for Slc11a1/Nramp1 which helps maintain iron homeostasis via phagolysosomal transport of divalent cations including iron [49], efficiently recycle erythrocyte-derived iron in macrophages [51], and are resistant to *S*. Typhimurium infection [19]. Indeed iron loading has been shown to increase susceptibility to acute *S*. Typhimurium infection in mice [23], therefore, iron deficiency may have contributed to increased bacterial resistance in our studies. In addition, Sv129 mice are better able to maintain iron homeostasis under conditions of both iron deficiency and excess compared to other mouse strains including C57BL/6, DBA/2 and CBA [50]. Wild type Sv129S6 mice have been shown to mobilize liver iron stores in response to iron deficiency where iron-limited erythropoiesis was maintained in the face of developing microcytosis [47]. Therefore, this Sv129S6 infection model may prove particularly useful in elucidating mechanisms of anemia in HLH, and altered iron homeostasis and anemia during chronic *S*. Typhimurium infection that are different from ACD.

Due to expansion of red pulp by EMH and inflammation, splenomegaly persists in mice even after bacterial CFU have cleared. Persistent splenomegaly also occurs in human HLH [2], and in prior studies of *S*. Typhimurium-infected mice [19], [31]. This mechanism may include persistence of bacterial antigen, for instance on follicular dendritic cells and/or on MHC class II molecules [22], [52], [53], that continues to drive cytokine production, inflammation and increased EMH in response to anemia and thrombocytopenia, resulting in persistent splenomegaly. Importantly, mice with the greatest magnitude of splenomegaly also had the most severe cytopenias.

Approximately 30% of the mice in our studies developed clinical neurological signs from which most recovered. The incidence of neurological disease in HLH patients varies from 37% in children [34], to 7–47% for all patients [3]. Neurological disease, while previously characterized in Slc11a1/Nramp1 wild type mice orally infected with *S.* Typhimurium [32], is clearly relevant to both HLH and typhoid fever, and may provide a means to clinically monitor response to therapeutic interventions.

Abnormal lipid processing is known to occur with HLH [5], [28], [54], and one of the diagnostic criteria for HLH is hypertriglyceridemia [5]. An effect on lipid processing in *S*. Typhimurium-infected mice is suspected based on finding vacuolated lymphocytes on blood films and foamy bone marrow macrophages (Figure 2B), features that also occur with lipid storage diseases [55], [56]. Cytokine-mediated decreases in lipoprotein lipase have been shown to occur with increased IFNγ and TNFα in mice [57], and TNFα in human fHLH patients [54]. In addition, a role for lipid (via cholesterol esterification) in intracellular survival of *S*. Typhimurium has been suggested [58]. A future direction for understanding HLH pathophysiology in this model will be investigating altered lipid metabolism including lipoprotein lipase, triglycerides, cholesterol and PGF2α analyses.

The important findings in highly infected mice in this chronic systemic infection model are those demonstrating first, an HLH syndrome and second, anemia different from ACD and acute murine *S.* Typhimurium infection. To understand how HLH develops and can be ameliorated, it is necessary to understand the molecular and cellular events that can precipitate HLH. A viable animal model is critical to understanding pathogenesis and for intervention strategies. Thus, future directions for this mouse model include cytokine profiling and characterization of specific lipid and iron cellular markers and metabolism. A further understanding of the unique pathologies of increased hemophagocytosis, hyperferritinemia and inflammatory disease without tissue iron loading, in the face of microcytic anemia may help elucidate pathophysiology of anemia in HLH that differs from ACD. Features of *S*. Typhimurium-infected mice that substantiate its value as an sHLH animal model include a natural host-pathogen interaction, no known single-locus genetic component, death is not the endpoint, a commercially available mouse strain and infectious agent, and a tissue-load dependent and manipulable model. *S*. Typhimurium-infected mice have the features of sHLH yet have a low mortality rate (approximately 7%) even without treatment intervention. This provides a viable model to study sHLH pathogenesis over time, and novel targets for HLH therapies and therapeutic response by clinical, hematological, immunological, pathological and molecular means.

## Materials and Methods

### Ethics Statement

Research protocols were in accordance with the NIH Guide for the Care and Use of Laboratory Animals, and approved by the University of Colorado Institutional Biosafety and Animal Care and Use Committees.

To characterize hematological responses and establish clinico-pathologic features of HLH in *S*. Typhimurium-infected mice, two studies of 16 and 10 weeks duration, respectively, were undertaken. Specific HLH diagnostic criteria [5] analyzed included hemophagocytic macrophages in bone marrow and spleen, fever, splenomegaly, cytopenias, plasma fibrinogen, and serum ferritin.

### Bacteria and Mice

Freshly struck colonies of virulent *Salmonella enterica* serotype Typhimurium strain SL1344 (StrR) [24], [59], inoculated into Luria-Bertani broth were grown at 37°C with aeration overnight. Bacteria were pelleted and resuspended in sterile PBS at 9.1×10^8^ – 2.0×10^9^ CFU in 100 µL, as determined by plating. Streptomycin (200 µg/mL) was added to media to select for *S*. Typhimurium. Female seven week-old Sv129S6/SvEvTac mice (Taconic Farms, Inc.; Hudson, NY) were fasted for 12–16 hours prior to infection, and housed separately from mock-infected control mice.

### 16-Week Experiment

#### Hematology and IgG enzyme-linked immunosorbent assay (ELISA)

Blood (50–150 µL) was collected bi-weekly by retro-orbital method into Microvette K_2_-EDTA tubes (Sarstedt, Inc; Newton, NC) for CBC using a Hemavet HV950FS analyzer (Drew Scientific; Waterbury, CT). Simultaneously, serum was collected from one mock-infected control and five infected mice for IgG to *Salmonella* O-Antigen analysis. Following euthanasia, whole spleen, liver, and MLN were weighed, homogenized and plated for bacterial CFU. Serum IgG to *Salmonella* O-Antigen, measured by anti-*Salmonella* IgG ELISA included an overnight culture of *S*. Typhimurium that was pelleted, washed with PBS, weighed and sonicated in PBS. Supernatant was harvested after one hour centrifugation at 4,000 g and diluted to 100 ng of bacteria per 100 µL. 100 µL of lysate was incubated overnight in 96-well ELISA plates (Nunc; Rochester, NY). Coated wells were washed and blocked (PBS with 0.05% Tween, 1% Bovine Serum Albumin) for one hr at 37°C. Sera were diluted serially in blocking buffer, incubated in blocked wells for two hours at room temperature, then washed. Anti-mouse IgG antibody conjugated to horseradish peroxidase (HRP) (Abcam ab6728-1; Cambridge, MA) was added (1∶4000), incubated for two hours at room temperature, washed and incubated with HRP substrate according to instructions (Pierce Protein Research Products, Rockford, IL). Absorbance was read at 450 nm with a plate reader. Serum titer was determined from the highest dilution of serum to give an optical density above pre-immune serum background.

### 10-Week Experiment

#### Body temperature and blood collection

The submandibular vascular bundle collection method replaced retro-orbital bleeding to minimize distress to the animal and allow for greater collection volume [60]. Approximately 300 µL of blood was collected into Microtainer K_2_-EDTA and serum tubes (BD; Franklin Lakes, NJ) for hematology and serum analyses at a single time-point in mice which were then euthanized at one, three, six and ten weeks post-infection. Blood kept at room temperature was analyzed within four hours of collection for all parameters except ferritin, which was frozen for later analysis. Compete blood counts were performed using mouse specific instrumentation on an ADVIA 120 hematology analyzer with Multispecies software (version 3.1.8.0-MS) (Bayer Corporation, Tarrytown, NY). Serum iron was measured with an Hitachi 917 analyzer (Roche Diagnostics, Indianapolis, IN). Plasma fibrinogen was measured by heat precipitation method. Fresh blood smears were Wright-stained, and manual leukocyte differential counts were performed to address increased percentages of large unstained cells (LUC) measured by the Advia 120 in seventeen of twenty infected mice (range 2.0–6.0%) compared to mock-infected controls (mean 1.0%). The majority of LUC (1–4%) were activated monocytes, the remainder were large lymphocytes; automated monocyte counts were corrected accordingly. Neurological signs and body temperatures were recorded at the same time daily, and body weights weekly. Five temperature measurements per mouse were made daily by digital infrared thermometer (MT-100; Micro Temp, Troy, MI) as previously described [61]. Mean difference between infected and mock-infected mice for each day was computed and tested in a one-sided paired *t* test to establish whether the difference was greater than zero. *P*<0.05 was considered significant (GAUSS Mathematical & Statistical System Version 8.0; Aptech Systems, Inc.; Black Diamond, WA).

#### Cyto- and histopathology

Following euthanasia whole spleen and liver were weighed; a section of each was weighed, homogenized and plated for bacterial CFU. No CFU were found in any mock-infected animals. Remaining liver and spleen, and one femur were collected into 10% neutral buffered formalin for histological evaluation. Bone marrow brush smear preparations were made immediately post-mortem from the second femur, and Wright-stained for cytological examination. Tissues were processed by routine histological methods; paraffin sections were stained with hematoxylin and eosin (H & E), Perl's Prussian blue for ferric iron (hemosiderin), and Masson's trichrome stain for fibrosis [62]. Intensity of iron tissue staining was subjectively scored on a scale of 0 – 4+ by light microscopy. Photomicrographs were taken with an Olympus BX50 (Olympus Corporation, Center Valley, PA) using NIS-Elements package F3.0 Nikon Laboratory Imaging software (Nikon Corporation, Tokyo, Japan).

#### Serum ferritin ELISA

Ferritin was quantified using a commercially available mouse ELISA kit (E-90F, Immunology Consultants Laboratory, Inc; Newberg, OR). Absorbance at 450 nm was determined with a plate reader; ferritin concentrations were calculated based on standard curves developed within the same plate and corrected for sample dilution. To verify findings, serum ferritin was measured in duplicated infection experiments using an additional 10 *S*. Typhimurium-infected and three mock-infected control mice at three weeks post-infection.

#### Flow cytometry and cell sorting

Spleens from mice collected three weeks after *S*. Typhimurium infection were mechanically homogenized and passed through a 70 µm cell strainer (BD Biosciences, San Jose, CA) to obtain single cell suspensions. Non-nucleated erythrocytes were lysed in hypotonic buffer containing 0.16 M NH4Cl, 10 mM KHCO3 and 0.1 mM EDTA. Non-lysed cells were passed through a 40 µm cell strainer and re-suspended in staining buffer (PBS +1% FBS and 0.02% sodium azide) containing anti-mouse CD16/32 (eBioscience, San Diego, CA) to block Fc receptors. For analysis of erythroblasts, cells were then stained with allophycocyanin-conjugated anti-mouse TER119 antibody [40] (BD Biosciences, San Jose, CA), followed by staining for DNA in 10 µg/mL DAPI (Invitrogen, Carlsbad, CA). Fluorescently labeled cells were quantified using a CyAn ADP flow cytometer (Beckman Coulter, Fulleton, CA) and analyzed using FlowJo software (Tree Star, Inc., Ashland, Or). For hemophagocytic macrophage sorting, cells were stained with phycoerythrin-conjugated anti-mouse CD11b and fluorescein isothiocyanate-conjugated anti-mouse GR1 antibodies (eBioscience, San Diego, CA). Splenocytes were fixed in 1% paraformaldehyde/1% sucrose and permeabilized in 0.1% saponin, then stained intracellularly for erythrocytes with allophycocyanin-conjugated anti-mouse TER119 antibody, followed by staining for DNA in 10 µg/ml DAPI. Cells were passed through another 40 µm cell strainer, then sorted for CD11b^high^, GR1^low^ macrophages with at least 6N DNA content using a MoFLoXDP cell sorter (Beckman Coulter, Fullerton, CA), and cytospun onto poly-L-lysine coated slides (Wescor, Logan, UT) for microscopic analysis. Confocal fluorescent micrographs were captured using a Yokogawa CSU10 spinning disk confocal (Tokyo, Japan) on a Nikon Eclipse TE2000-U inverted microscope (Tokyo, Japan) with a 100× Plan Apo numerical aperture 1.4 objective lens, and acquired with a Cascade II:512 (Photometrics, Tucson, AZ) camera using MetaMorph 7.0 software (Molecular Devices, Sunnyvale, CA).

#### Statistics

Mann-Whitney U Test and Student's *t*-Test were performed and considered significant at *P*<0.05. Results from both tests were similar; only *t*-Test results are shown.
